# On-Chip Photon Angular Momentum Absolute Measurement Based on Angle Detection

**DOI:** 10.3390/nano12050847

**Published:** 2022-03-02

**Authors:** Houquan Liu, Zhenghao Xie, Jiankang Xu, Libo Yuan

**Affiliations:** 1 Photonics Research Center, School of Optoelectronic Engineering, Guilin University of Electronic Technology, Guilin 541004, China; xiezh1003@foxmail.com (Z.X.); jiankangx3@gmail.com (J.X.); lbyuan@guet.edu.cn (L.Y.); 2 Guangxi Key Laboratory of Optoelectronic Information Processing, Guilin University of Electronics Technology, Guilin 541004, China; 3 Guangxi Key Laboratory of Automatic Detecting Technology and Instrument, Guilin University of Electronics Technology, Guilin 541004, China

**Keywords:** photon angular momentum, surface plasmon polaritons, on-chip photon device

## Abstract

Photon angular momentum (AM) has been widely studied due to its unique properties. The accurate detection of photon AM is very important in its wide applications. Though various on-chip AM detectors based on surface plasmon polaritons (SPPs) have been proposed, most of them can only realize relative measurement. For example, most photon orbital angular momentum (OAM) detectors measure the high order OAM via measuring the relative interval between the intensity spots of the SPPs excited by the target order OAM beam and the reference order (usually 0th order) OAM beam. In this paper, we propose a simple on-chip photon AM detector. It can realize absolute measurement of photon OAM via angle detection, whose measurement result does not depend on the measurement of any reference OAM beam. At the same time, it can also recognize photon spin angular momentum (SAM). The proposed detector provides a new way for absolute measurement of photon AM, which may have some potential applications in the field of integrated photonic device.

## 1. Introduction

Light is an electromagnetic wave, which carries not only energy information, but also momentum information. The momentum of light can be divided into linear momentum and angular momentum. Photon angular momentum includes spin angular momentum (SAM), determined by polarization state, and orbital angular momentum (OAM), determined by phase distribution [1]. Due to the unique properties of SAM and OAM, photon angular momentum has been widely applied in basic physics, applied physics, and biomedicine. For example, chiral nanomaterials can be designed for chirality analysis; binary encoding and data transmitting can be realized by using left-hand and right-hand polarization state in optical communication; higher dimensional information communication can be achieved by using the orthogonality of different order OAM beams [2,3,4,5]; and micro particles and cells can be rotated by angular momentum for optical manipulation [6,7]. Due to its wide application in many fields, the accurate detection of photon angular momentum is of great meaning and value.

In traditional optics, the SAM can be detected by polarizers, and the OAM can be distinguished by the number of branches of the interference fringes produced by the interference of the reference beam and vortex beam [8]. Although these methods can achieve accurate measurement of SAM and OAM, they all rely on optical elements with huge footprint. In recent years, as a new technology, surface plasmon polaritons (SPPs) provide a powerful tool for realizing on-chip integrable detector of SAM and OAM [9]. Through etching specially designed nano-structures on metal films, the SAM and OAM can be detected by using the SPPs generated from the coupling of input light and these structures [10,11,12,13,14,15,16,17,18]. For example, via the coupling of the OAM beam and semi-circular nano groove structure on metal film, an SPP’s intensity spot will be generated and the position of the intensity spot will shift when the OAM varies; hence, the OAM can be distinguished according to the shift distance of the intensity spot [15] Via the coupling of OAM beam and parallel nano slit structure, the directional launched SPPs beam can be generated according to the OAM; hence, the OAM can be distinguished according to the launching direction of the SPPs beam [16,17]. By combining the mechanism of detecting SAM and OAM in the same structure, the SAM and OAM can even be detected at the same time [19,20,21,22]. These schemes undoubtedly greatly reduce the volume of photon angular momentum detectors and improve system integration. However, these methods can only realize relative measurement; for example, the photon OAM detectors measure the high order OAM via measuring the relative interval between the intensity spots of the SPPs excited by the target order OAM beam and the reference order (usually 0th order) OAM beam; and the study of chip integrated methods for absolute OAM measurement is lacking. In our opinion, there exist at least two deficiencies in the relative measurement methods. One is the need to calibrate the detector with a reference OAM beam before measuring the target order OAM beam. The other one is that the measurement result can be affected by the accuracy of the scale amplification factor of the microscopic imaging system in measuring the relative interval between the SPPs intensity spots excited by the target and reference OAM beams. Though accurate interval measurements can be achieved easily in a laboratory by using commercial microscope, the need for accurate calibration of the scale amplification factor of a highly integrated microscopic imaging system will undoubtedly increase the complexity and difficulty of practical applications.

In this paper, we propose a simple on-chip photon angular momentum detector. It can realize absolute measurement of OAM via angle detection, whose measurement result is not affected by the amplification factor and does not depend on any reference OAM beam. At the same time, it can also recognize photon SAM. It provides a new way for absolute measurement of photon angular momentum.

## 2. Investigation and Discussion

**Figure 1:**
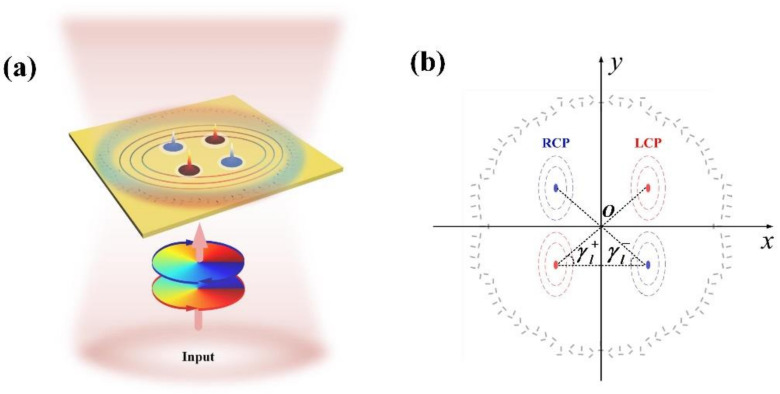
(**a**) The schematic and (**b**) coordinate system of our on-chip photon angular momentum detector. In (**a**), the two blue and red peaks represent the two SPPs’ intensity spots excited by RCP and LCP OAM beams, respectively. In (**b**), the two blue dots surrounded by blue dashed lines and the two red dots surrounded by red dashed lines represent the two SPPs’ intensity spots excited by RCP and LCP OAM beams, respectively. $\gamma_{l}^{-/+}$ is the angle between the connecting line of two SPPs intensity spots and *x*-axis.

**Figure 2:**
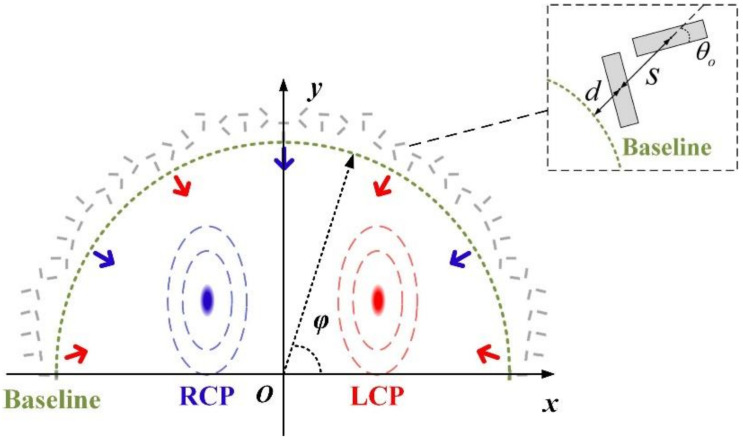
Details of the arrangement of orthogonal nano slit pairs in semi-annular array. The green dotted line is an annular baseline, the red and blue arrows indicate the propagation direction of the SPPs’ wave converging toward the center, *s* is the interval between the center points of the two slits of a pair, *d* is the interval between the baseline and the center point of inside slit (close to coordinate center), *θ_o_* is the angles between the outside slit (far away from coordinate center) of the pair and the radial direction, and φ is the azimuth angle of the polar coordinate system.

The schematic diagram of our on-chip photon angular momentum absolute measurement detector is shown in Figure 1. In our detector, orthogonal nano slit pairs arranged according to specific rule are etched on the thin gold film deposited on the silicon dioxide substrate. The input beam with different SAM and OAM illuminates the structure normally from the substrate side. Via the coupling of the input light and the nano slits pairs, the SPPs can be excited, which will propagate on the surface of gold film [23]. The SPPs distribution on the gold film surface can be controlled by adjusting the spatial distribution of the nano slits. Via properly designing the arrangement rule of the nano slit pairs, it can be realized that a beam with a specific OAM and SAM can excite two SPPs’ intensity spots. The two blue and red peaks in Figure 1a represent the two SPPs’ intensity spots excited by two OAM beams with different SAM respectively. Similarly, the two blue dots surrounded by blue dashed lines represent the two SPPs’ intensity spots excited by right-handed circularly polarization (RCP) OAM beam, and the two red dots surrounded by red dashed lines represent the two SPPs’ intensity spots excited by left-handed circularly polarization (LCP) OAM beam. The angle between the connecting line of the two excited SPPs’ intensity spots and *x*-axis is represented by $\gamma_{l}^{-/+}$, where the superscript −/+ indicates that the input polarization is RCP/LCP, and the subscript *l* indicates the order, i.e., the topological charge of the input OAM beam $E_{input}\propto \exp\left(il\varphi \right)$, where φ is the azimuth angle of the polar coordinate system shown in Figure 2.

In our design, when the polarization of the OAM beam is RCP, the two SPPs’ intensity spots are, respectively, located in the second and fourth quadrants of the rectangular coordinate system, and when the polarization is LCP, the two SPPs’ intensity spots are, respectively, located in the first and third quadrants. Hence, the SAM of the input beam can be first distinguished according to the location sides of the SPPs’ intensity spots. Then, when the light OAM varies, the positions of the SPPs’ intensity spots will shift, which leads to the change of the angle $\gamma_{l}^{-/+}$. Therefore, the OAM can be next determined by measuring $\gamma_{l}^{-/+}$. As $\gamma_{l}^{-/+}$ is the angle relative to *x*-axis, it can realize absolute measurement of the OAM without any additional reference beam, and the measurement result will not be affected by the amplification factor of the light intensity measurement system.

To show the details of our design, we first introduce the result of [20], where orthogonal nano slit pairs arranged in semi-annular array are used for simultaneous detection of light SAM and OAM. Figure 2 gives the details of the arrangement of orthogonal nano slit pairs in semi-annular array, in which the green dotted line is an annular baseline, the red and blue arrows indicate the propagation direction of the SPPs’ wave converging toward the center, the nano slit pairs are arranged along the baseline, *s* is the interval between the center points of the two slits of a pair, *d* is the interval between the baseline and the center point of inside slit (close to coordinate center), *θ_o_* is the angles between the outside slit (far away from coordinate center) of the pair and the radial direction, and φ is the azimuth angle of the polar coordinate system. When this semi-annular nano slit array structure is excited by the input beam, at the position of the baseline, the complex amplitude of the SPPs contributed from the inside slit and the outside slit of a pair located at azimuth angle φ can be expressed as

(1) $$\begin{array}{l} A_{i}^{\pm }\propto \cos\left(\theta_{o}\right)\exp[\pm i\left(\varphi -\theta_{o}\right)]\exp\left(ik_{SPP}d\right)e^{if\left(\varphi \right)},\\ A_{o}^{\pm }\propto \sin\left(\theta_{o}\right)\exp[\pm i\left(\varphi -\theta_{o}+\pi /2\right)]e^{ik_{SPP}\left(d+s\right)}e^{if\left(\varphi \right)}, \end{array}$$

respectively, where $k_{SPP}=2\pi /\lambda_{SPP}$ is wave vector with *λ_spp_* being the wavelength of the SPPs wave, $f\left(\varphi \right)$ is the phase distribution of the input beam (if the input beam is *l* order OAM beam, we have $f\left(\varphi \right)=l\varphi$, and superscript −/+ indicates that input polarization is RCP/LCP. Hence, under the case of *s* = *λ_spp_*/2, the total complex amplitude of the SPPs is

(2) $$A^{\pm }=A_{o}^{\pm }+A_{i}^{\pm }\propto e^{\pm i\varphi }e^{\mp i2\theta_{o}}e^{ik_{SPP}d}e^{if\left(\varphi \right)}.$$

In Equation (2), the $\theta_{o}$ and *d* dependent phase profile of $A^{\pm }$ is given by

(3) $$\varphi_{+}=k_{SPP}d-2\theta_{o};\varphi_{-}=k_{SPP}d+2\theta_{o}.$$

It can be seen that the phase profile of the SPPs’ wave at the baseline can be controlled by controlling $\varphi_{+}$ and $\varphi_{-}$, which can be realized by designing the parameters *d* and *θ_o_* of each nano slit pairs precisely. Then SAM dependent wavefront control of the SPPs’ wave at the annular baseline can be achieved. If the phase distribution of $\varphi_{+}$ is designed so that the phase difference between the SPPs’ waves excited from each slit pair is zero when they propagate to the point ($x_{L}$, $y_{L}$), the SPPs’ waves will focus to this point, i.e., the semi-annular nano slit array structure will generate a SPPs’ intensity spot at ($x_{L}$, $y_{L}$) when the input polarization is LCP. Similarly, if the phase distribution of $\varphi_{-}$ is designed so that the phase difference between the SPPs’ waves excited from each slit pair is zero when they propagate to the point ($x_{R}$, $y_{R}$), the semi-annular nano slit array structure will generate an SPP intensity spot at ($x_{R}$, $y_{R}$) when the input polarization is RCP. Moreover, it is found that if the OAM of the incident beam varies, the positions of the two SPPs’ intensity spots will shift. Hence, the location of the SPPs intensity spots can be used to distinguish the incident polarization, and the shift of the intensity spots can be used to measure the OAM [20]. To show this more clearly, we design appropriate phase profiles of $\varphi_{+}$ and $\varphi_{-}$, and then perform some finite-difference time-domain (FDTD) simulations to calculate the SPPs intensity distributions.

**Figure 3:**
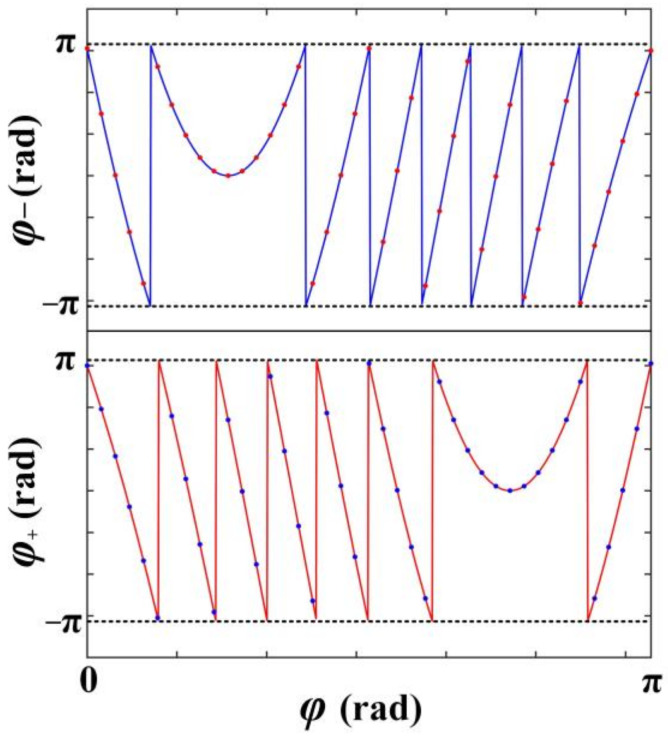
The phase profiles of *φ*_+_ and *φ*_−_ for a semi-annular nano slit array structure to generate a SPPs intensity spot at (2.3 μm, 2.5 μm) when the input polarization is LCP and a SPPs intensity spot at (−2.3 μm, 2.5 μm) when the input polarization is RCP. The blue and red solid lines represent continuous phase profiles of *φ*_+_ and *φ*_−_, respectively. The red and blue dots represent the sampling points on the phase profiles.

**Figure 4:**
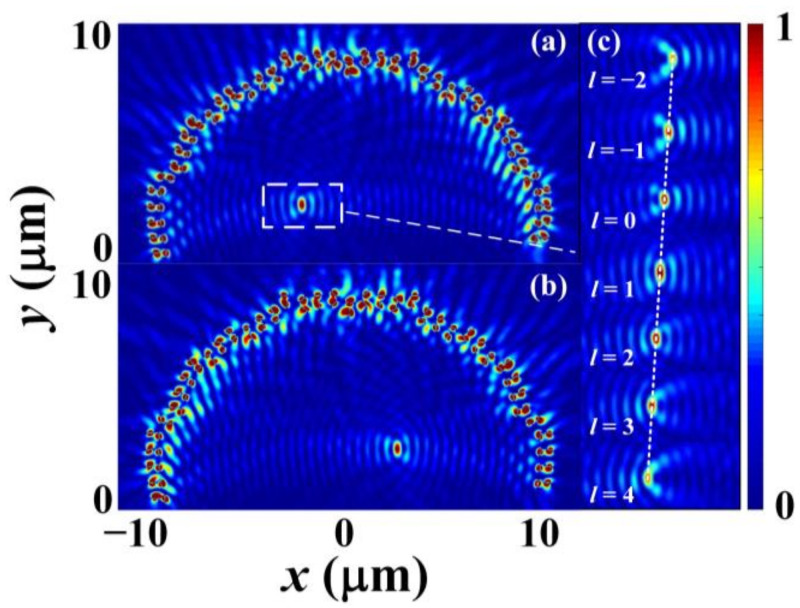
(**a**) Normalized intensity of the SPP’s field excited by RCP incidence light. The SPP’s intensity spot is framed by a white dotted line. (**b**) Normalized intensity of the SPP’s field excited by LCP incidence light. (**c**) Numerical simulations results of the shift of the SPP’s intensity spot when the incidence light has RCP and different OAM. The white dotted line is used to connect the SPPs’ intensity spots under different *l*.

The phase profiles shown in Figure 3 are designed to achieve ($x_{R}$, $y_{R}$) = (−2.3 μm, 2.5 μm) and ($x_{L}$, $y_{L}$) = (2.3 μm, 2.5 μm) for incident wavelength of *λ*_0_ = 980 nm, which determines *λ_spp_ = λ*_0_$[(\varepsilon_{d}+\varepsilon_{m}^{\prime })/\varepsilon_{m\;}^{\prime }\varepsilon_{d}]$^0.5^ = 970 nm and *s* = 485 nm, where $\varepsilon_{m}^{\prime }$ is the real part of the relative permittivity of the gold and $\varepsilon_{d}$ is the relative permittivity of air. In a real nano slit array structure, generating continuous phase profiles of $\varphi_{+}$ and $\varphi_{-}$ shown in Figure 3 is not achievable, and one slit pair can only control the phase of one sampling point on the phase profiles. Therefore, we build our FDTD simulation model (using Lumerical FDTD Solutions) to simulate the SPPs intensity distribution via evenly sampling 40 sampling points on the phase profiles as shown by the red and blue dots in Figure 3. The simulation results are shown in Figure 4, where Figure 4a,b are the normalized intensity of SPP fields excited by RCP and LCP incidence light respectively, and Figure 4c shows the shift of the SPPs’ intensity spots when the incidence light has RCP and different OAM. It should be noted that throughout this paper, the radius of the baseline, the thickness of the gold film, and the length, width, and depth of the nano slits are set to be 10 um, 150 nm, 400 nm, 100 nm, and 150 nm, respectively.

**Figure 5:**
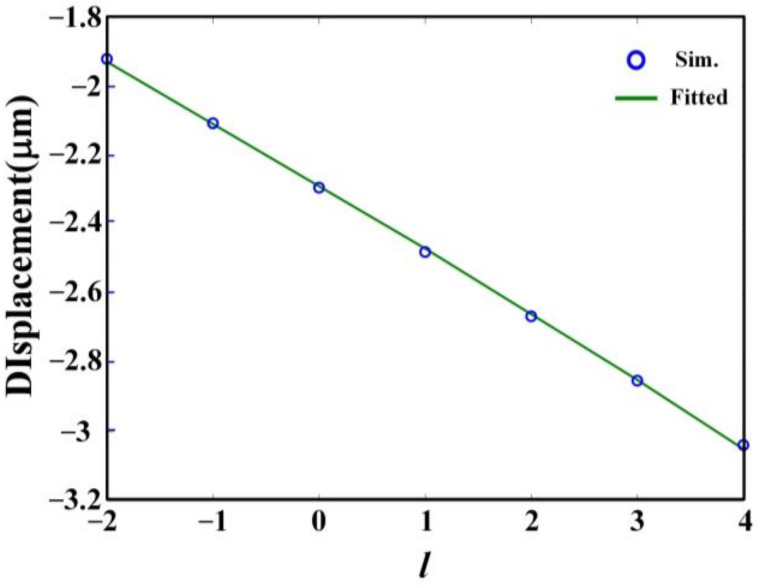
The horizontal positions, i.e., the *x*-axis coordinate value of the SPPs intensity spots when the incidence light has RCP and different OAM. The horizontal axis *l* is the topological charge of the OAM beam. The blue circular markers are the simulation results. The green solid line is fitted curve of the simulation results.

From the results, it can be seen clearly that a semi-annular nano slit array structure can generate an SPP intensity spot at (−2.3 μm, 2.5 μm) when the input polarization is RCP and an SPP intensity spot at (2.3 μm, 2.5 μm) when the input polarization is LCP. When the incidence light possesses OAM, the SPPs’ intensity spots will shift as the OAM varies. Figure 5 further gives the horizontal positions, i.e., the *x*-axis coordinate value of the SPPs intensity spots when the incidence light has RCP and different OAM. In the figure, the blue circular markers are the FDTD simulation results, and the green solid line is the fitted curve of the simulation results. The function of the fitted curve is *D* = −0.1861*l* − 2.294, which implies that the SPPs intensity spot will shift a lateral displacement of about 0.19 μm when the change of the topological charge is 1.

The results of Figure 4 and Figure 5 show clearly that simultaneous detection of light SAM and OAM can be achieved by designing a semi-annular nano slit array. This is the main result of [20]. However, in this semi-annular nano slit array structure, only one SPP intensity spot located in the second quadrant is excited when the input polarization is RCP, and only one SPP intensity spot in the first quadrant is excited when the input polarization is LCP. In this case, due to the lack of additional reference points, absolute measurement of the OAM is not achievable. To measure a target OAM beam, an additional reference point should be provided by using the SPP’s intensity spot excited by the 0th order OAM beam, and then the target OAM is determined by the interval between the two SPPs’ intensity spots excited by the 0th and target order OAM beams, whose accurate measurement relies on the accurate calibration of the scale amplification factor of the microscopic imaging system, which will increase the complexity and difficulty of practical applications. In spite of this, the results of [20] inspired our absolute measurement scheme. If we design a symmetrical semi-annular nano slit array structure below the *x*-axis, we believe that the expected function of the absolute measurement detector can be realized, i.e., when exciting the total structure (the final annular nano slit array structure) by a beam with specific OAM and SAM, two SPP intensity spots can be generated, and the two SPPs’ intensity spots are, respectively, located in the second and fourth quadrants when the polarization is RCP, and the two SPP intensity spots are, respectively, located in the first and third quadrants when the polarization is LCP. This is the major ideal of this paper. In the following, we demonstrate that the expected function is achievable on the symmetrical annular nano slit array structure by FDTD simulation, and show that it can be used to realize absolute measurement in details.

**Figure 6:**
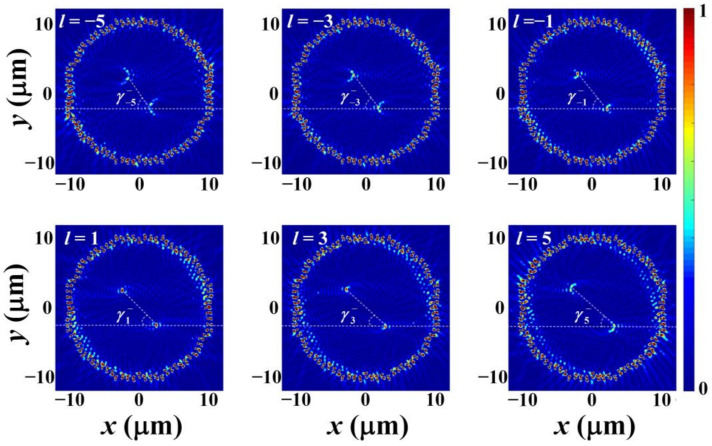
The FDTD simulated normalized SPP intensity distributions under topological charge *l* = −5, −3, −1, 1, 3, and 5 when the incidence polarization is RCP. The white dotted lines are used to connect the two SPPs’ intensity spots.

**Figure 7:**
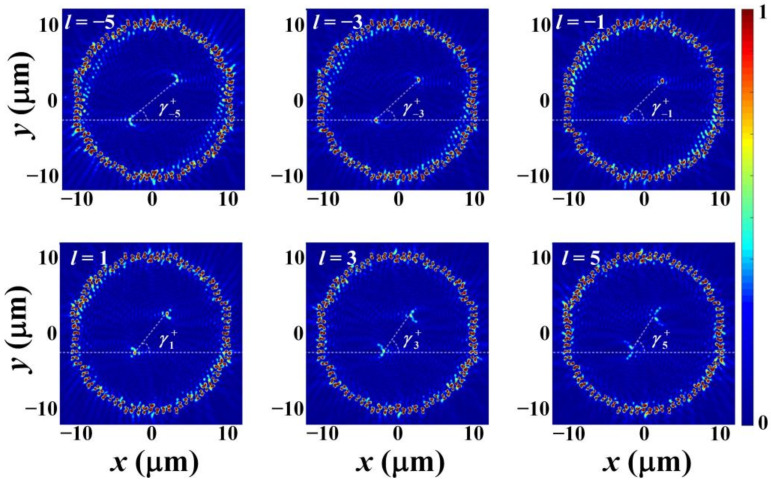
The FDTD simulated normalized SPP intensity distributions under topological charge *l* = −5, −3, −1, 1, 3, and 5 when the incidence polarization is LCP. The white dotted lines are used to connect the two SPPs’ intensity spots.

**Figure 8:**
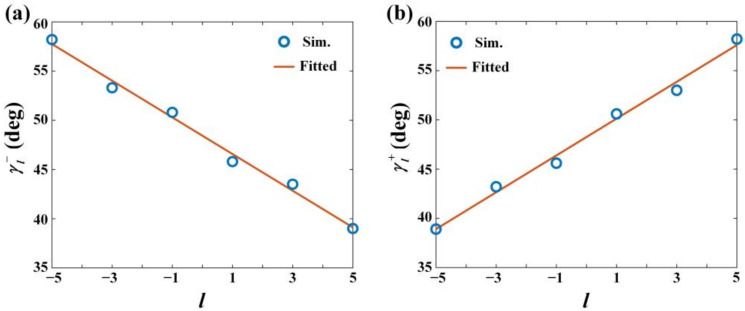
(**a**) The simulation results of $\gamma_{l}^{-}$ and their fitted curve when the incidence polarization is RCP and (**b**) the simulation results of $\gamma_{l}^{+}$ and their fitted curve when the incidence polarization is LCP, where the horizontal axis is the value of topological charge *l*, and the vertical axis is $\gamma_{l}^{-}$ and $\gamma_{l}^{+}$, respectively. The blue circular markers are the simulation results. The orange line is fitted curve of the simulation results.

Figure 6 and Figure 7 are the simulated normalized SPP intensity distributions under *l* = −5, −3, −1, 1, 3, and 5 when the incidence polarization is RCP and LCP, respectively. In the simulation, the semi-annular nano slit arrays above and below the *x*-axis are symmetrical, and the other simulation parameters are same as that used in Figure 4. From the figures, as expected, it is clear that two SPP intensity spots are generated and located in the second and fourth quadrants, respectively, when the polarization is RCP, and two SPP intensity spots are generated and located in the first and third quadrants, respectively, when the polarization is LCP. Moreover, with the increase of *l*, when the polarization is RCP, the SPP intensity spot in the second quadrant will shift along the negative direction of *x*-axis and the SPP intensity spot in the fourth quadrant will shift along the positive direction of *x*-axis, which causes the decrease of $\gamma_{l}^{-}$. The simulated $\gamma_{l}^{-}$ for *l* = −5, −3, −1, 1, 3, and 5 are 58.2 deg, 53.3 deg, 50.8 deg, 45.8 deg, 43.5 deg, and 39.0 deg, respectively. These results and their fitted curve are shown in Figure 8a. The function of the fitted curve is $\gamma_{l}^{-}$ = −1.863 *l* + 48.43, implying the change of $\gamma_{l}^{-}$ is about 1.86 deg when the change of the topological charge is l. Similarly, with the increase of *l*, when the polarization is LCP, the SPP intensity spot in the first quadrant will shift along the negative direction of *x*-axis and the SPP intensity spot in the third quadrant will shift along the positive direction of *x*-axis, which causes the decrease of $\gamma_{l}^{+}$. The simulated $\gamma_{l}^{+}$ for *l* = −5, −3, −1, 1, 3, and 5 are 38.9 deg, 43.2 deg, 45.6 deg, 50.6 deg, 53.0 deg, and 58.2 deg, respectively. These results and their fitted curve are shown in Figure 8b. The function of the fitted curve is $\gamma_{l}^{+}$ = 1.87*l* + 48.25, implying the change of $\gamma_{l}^{+}$ is about 1.87 deg when the change of the topological charge is l. Obviously, the SAM of the input beam can be distinguished according to the quadrants that the SPPs’ intensity spots locate in and the OAM can be determined by measuring $\gamma_{l}^{-/+}$. It should be noted that when |*l*| > 5, the SPP intensity spot will show crescent-shaped distribution which will affect the precise determination of its position. Hence, the actual effective detection range of the OAM is |*l*| ≤ 5.

**Figure 9:**
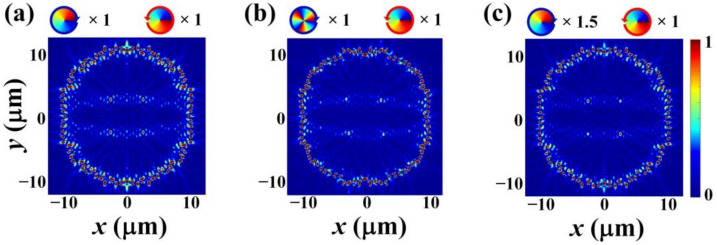
Simulation results of the SPPs’ intensity distributions under exciting by different mixed light with different SAM and OAM. (**a**) The SPPs’ intensity distribution excited by mixed light including +1 order RCP OAM beam and −1 order LCP OAM beam with equal amplitude. (**b**) The SPPs’ intensity distribution excited by mixed light including +3 order RCP OAM beam and −1 order LCP OAM beam with equal amplitude. (**c**) The SPPs’ intensity distribution excited by mixed light including +1 order RCP OAM beam with relative amplitude of 1.5 and −1 order LCP OAM beam with relative amplitude of 1.

In above discussions, we have shown that the designed annular nano slit array structure can realize absolute measurement of the angular momentum of a beam possessing single order of OAM by angle measurement. In the following, we will numerically demonstrate that our detector can be further expanded to realize angular momentum detection of mixed light with different SAM and OAM. Figure 9 shows the simulation results of the SPPs intensity distributions under exciting by different mixed light with different SAM and OAM, in which the two inserts above each intensity distribution are phase maps of the OAM beams mixed in the input light, the rings with arrow surrounding the phase maps indicate the polarizations of the corresponding OAM beams, and ×*m* represents that the relative amplitude of the corresponding OAM beam is *m*. Figure 9a is the SPP intensity distribution excited by mixed light including +1 order RCP OAM beam and −1 order LCP OAM beam with equal amplitude. The angles extracted from Figure 9a are $\gamma_{1}^{-}$ = 45.6 deg and $\gamma_{1}^{+}$ = 45.9 deg. Figure 9b is the SPPs intensity distribution excited by mixed light including +3 order RCP OAM beam and −1 order LCP OAM beam with equal amplitude. The angles extracted from Figure 9b are $\gamma_{3}^{-}$ = 43.6 deg and $\gamma_{-1}^{+}$ = 46 deg. Figure 9c is the SPP intensity distribution excited by mixed light including +1 order RCP OAM beam with relative amplitude of 1.5 and −1 order LCP OAM beam with relative amplitude of 1. The angles extracted from Figure 9c are $\gamma_{1}^{-}$ = 45.6 deg and $\gamma_{-1}^{+}$ = 45.6 deg. Compared to the results of Figure 6 and Figure 7, the extracted angles from Figure 9a–c exhibit an error value Δ$\gamma_{l}^{-/+}$ ≤ 0.4 deg, which may cause by two reasons: one is the error introduced in locating the position of the SPP intensity spots manually when we measure the angles, and the other one is that the presence of the other OAM beam will slightly affect the field distribution of the SPP intensity spots of the target OAM beam. In spite of this, the slight error value does not affect our correct identification of the OAM since it is significantly less than the angle change corresponding to the topological charge change of l. Therefore, our detector can achieve detection of SAM and OAM both when the input light possesses single order of OAM and when the input light is mixed light with different SAM and OAM. Further, comparing the results of Figure 9a–c, it can be found that the position of the SPPs’ intensity spots can be controlled by controlling the order of the input OAM beam, and the intensity of the SPPs’ intensity spots can be controlled by controlling the amplitude of the input OAM beam. Hence, beside measuring angular momentum, our detector can also be used for controlling the position and intensity of the SPPs’ intensity spots, which may have potential applications in optical manipulation.

## 3. Conclusions

In conclusion, a simple on-chip photon angular momentum absolute measurement detector is proposed. The detector consists of an annular nano slit array structure. When the detector is excited by RCP OAM beam, two SPP intensity spots will be generated, locating in the second and fourth quadrants, respectively; and when the detector is excited by an RCP OAM beam, two SPPs intensity spots will be generated, locating in the first and third quadrants, respectively. Hence, the SAM can be distinguished according to the quadrants that the SPPs’ intensity spots locate in. When the OAM of the input beam varies, the positions of the SPPs’ intensity spots will shift, leading to the change of the angle of the connecting line of the two excited SPP intensity spots and *x*-axis. Hence, the OAM can be determined by measuring the angles. Moreover, our detector can achieve detection of angular momentum, both when the input light possesses single order of OAM, and when the input light is mixed light with different SAM and OAM. Our result provides a new way for realizing photon angular momentum absolute measurement. It may have some potential applications in the field of the on-chip photonic device.

## Data Availability

All data are included in this letter.
